# *Foxp3* (−/ATT) Polymorphism Contributes to the Susceptibility of Preeclampsia

**DOI:** 10.1371/journal.pone.0059696

**Published:** 2013-04-01

**Authors:** Ximing Chen, Ting Gan, Zhiqiong Liao, Shengqiang Chen, Jianhua Xiao

**Affiliations:** 1 Medical College of University of South, Guangzhou, China; 2 The Third Affiliated Hospital of Guangzhou Medical College, Guangzhou, China; 3 The Second Affiliated Hospital of Guangzhou Medical College, Guangzhou, China; Institute of Zoology, Chinese Academy of Sciences, China

## Abstract

**Objective:**

To evaluate the potential influence of *Foxp3* polymorphism on preeclampsia (PE) susceptibility, we conducted a case-control study in Han Chinese women.

**Methods:**

*Foxp3* genotyping was determined by polymerase chain reaction with sequence-specific primers (PCR-SSP) in 156 PE patients and 252 age-frequency matched controls. Immunohistochemical staining was used to detect the expression of Foxp3 specific transcription factor in 30 PE and 30 normal pregnant women.

**Results:**

The positive rate of Foxp3 expression in PE (26.67%) was significant difference from that in normal control (63.33%, *P<*0.05). The frequency of *Foxp3*-6054 TT genotype was significantly lower in PE patient than that in control. No significant difference was found in *Foxp3*-3279 genotypes between PE and control, as well as for the variant allele. The frequency of *Foxp3*-6054A/−3279C haplotype in PE was significantly higher than that in control (P<0.01), while the frequency of *Foxp3* 6054T/−3279C haplotype was significantly lower in PE patient than that in control (P<0.01).

**Conclusion:**

Our findings suggest that the immune suppression function in PE patients is weakened, which may result in the occurrence of PE. *Foxp3* polymorphism (rs5902434) may be a potential contributor for the development of PE in Han Chinese women.

## Introduction

Preeclampsia (PE) is a severe complication of pregnancy with a worldwide incidence of 2-10%. It is also one of the leading causes of maternal and perinatal morbidity and mortality. PE has been associated with insufficient trophoblast invasion of maternal spiral arteries, impaired placental perfusion, and widespread endothelial cell dysfunction [1]–[3]. The causes leading to these pathological alterations remain unclear. Emerging evidence suggests that an excessive maternal inflammatory response to cytokinemediated endothelial damage during pregnancy plays a crucial role in PE pathogenesis.

Normal pregnancy requires a relative maternal immune tolerance for the fetus. It is known that women may increase risk of PE in the first pregnancy with the new partners. Decreased exposure to semen from the father of the child and preconception also increased risk of PE. Taken together, these observations indicated that PE may partially mediate by immune system. Immune suppression is a key function of regulatory T (Treg) cells [3], [4], which acts by limiting antigen-specific immune responses and thus gives rise to suppressed immune response to cancer cell [5]. Treg cells (Tregs) are defined by the expression of forkhead family transcription factor forkhead box p3 (*Foxp3*), a gene that maps to the short arm of X chromosome (Xp11.23) [6], [7]. Tregs are a subset of T-cells that are immunosuppressive. The expression of Foxp3 is required for Tregs development. Decreased numbers of circulating Tregs have been observed in women with pregnancy complications, including recurrent pregnancy loss and PE [5]–[9]. Tregs are produced by upregulation of Foxp3 expression that results in the conversion of naïve T cells to Tregs. Less is known about the function of *Foxp3* gene in humans [10]. It has been demonstrated in mouse models that inactivation of Foxp3 results in a deficiency of Tregs, and notable organ specific Foxp3 is a major regulator for the development and function of Tregs [11]. Deficiency of *Foxp3* may impair the suppressive function of Tregs [12]. Several previous studies have found an association between *Foxp3* gene polymorphisms and autoimmune diseases, such as systemic lupus erythematosus (SLE) [13], autoimmune thyroid diseases (AITDs) [14], type I diabetes (TID) [15], and allergic rhinitis [16]. The decreasd expression of Foxp3 in preeclampsia demonstrates that the reduction of Tregs numbers may result in the imbanance of immunologic tolerance between the mother and fetus,thereby participating in the pathogenesis of preeclampsia. However, the association between *Foxp3* polymorphisms and PE has not been reported so far. We undertook a case-control study to determine whether two genetic polymorphisms (rs5902434 del/ATT and rs3761548 A/C) in *Foxp3* gene are associated with PE risk.

## Materials and Methods

### Subjects

All subjects were recruited from the Department of Obstetrics and Gynecology at the second and the third affiliated hospitals of Guangzhou Medical College during Oct. 2011 and June 2012. The study subjects included women who subsequently developed PE (n = 156) and healthy pregnant women (n = 252) as controls. Normal pregnancy women were randomly selected from the contemporaneous women who were normotensive, without proteinuria throughout the pregnancy, and delivering a healthy neonate at term (>37 weeks of gestation) with no medical or obstetric complications, such as chronic hypertension, diabetes, renal insufficiency, congenital anomalies, intrauterine growth restriction (IUGR), or fetal demise. PE was defined as hypertension (systolic blood pressure ≥140 mmHg and/or diastolic blood pressure ≥90 mmHg) and proteinuria (≥300 mg in a 24-hr urine collection and/or ≥1+ on dipstick testing) after 20 weeks of gestation according to the Committee Terminology of the American College of Obstetricians and Gynecologists. Severe PE was defined as systolic blood pressure ≥160 mmHg and/or diastolic blood pressure ≥110 mmHg; severe proteinuria (urinary protein excretion ≥2.0 g per 24-hr and/or ≥2+ on dipstick testing); evidence of pulmonary edema; seizures; oliguria (<500 mL/day); thrombocytopenia (platelet count <100×10^9^/L); and severe central nervous system symptoms, such as altered mental status, headaches, blurred vision, or blindness. PE patients were divided into two subgroups: (1) mild (n = 43) and severe (n = 113) in order to analyze the differences by disease severity. Exclusion criteria included major congenital anomalies; prior PE; taking drugs; alcohol consumption; smoking; and preexisting medical conditions, such as diabetes, chronic hypertension, autoimmune disease, or renal disease. The Ethics Committee at the third affiliated hospitals of Guangzhou Medical College approved the use of the clinical information and the collection of samples for the current study. Written informed consent was obtained from all enrolled subjects.

### Genotyping

Genomic DNA was extracted from peripheral white blood cells and stored at –20°C until genotyping. Two SNPs (rs5902434 del/ATT and rs3761548) in *Foxp3* gene were analyzed by PCR-SSP assay. The primers included *Foxp3*-3279: F1 5′-CTGGCTCTC TCCCCAACTGA-3′, F2 5′- CTGGCTCTCTCCCCAACTGC -3′, R 5′-ACAGAGCC CATCATCAGACTCTCTA-3′. Foxp3-6054: F1 5′-ACCTTTAAGTCTTCTGCCATT TATTCTATTATTT- 3′,F2 5′- CCTTTAAGTCTTCTGCCATTTATTCCTATTAT TA-3′; R 5′-TGATTATCAGCGCACACACTCAT-3′. PCR amplification was performed with a total of 37.5 µL reaction mixture containing 40 ng DNA, 0.2 µM of each primer, 0.2 µM dNTP, 2.0 Mm MgCl_2_ and 1.25 U Taq DNA polymerase with 1×reaction buffer (Fermentas Inc., Canada). The PCR protocol included an initial denaturation step at 95°C for 5 min; 30 cycles of 1 min denaturation step at 95°C, 1 min annealing step at 58.6°C and 1 min extension step at 72°C, and with a 7 min final extension step at 72°C. The 442 bp and 333 bp PCR product was separated on a 2% agarose gel. The genotyping was obtained by PCR-SSP analysis and the accuracy of the PCR-SSP results was confirmed by DNA sequencing. The genotyping was performed by technicians blind to the subjects’ case control status. Ten percent samples were genotyped twice to ensure the quality of experiments. The reproducibility was 100%.

### Immunohistochemistry

The placenta were cut into small blocks, washed several times with phosphate buffered saline to remove excess blood, fixed for 12–16 h in neutral buffered formalin, and embedded in paraffin. Immunohistochemistry was performed using an avidinbiotin peroxidase method. Sections (5 µm) were cut from the paraffin-embedded tissue blocks and placed onto APES-coated slides. Each specimen was stained with H&E for histological analysis. Sections were stained with antibodies directed against human Foxp3 (Abcam, Cambridge, UK). Each paraffin section was deparaffinized, followed by antigen retrieval with epitope retrieval solution (10 mmol citrate buffer, pH 6.0) in a pre-heated water bath at 98°C for 10 minutes, and endogenous peroxidase was blocked by using 3% hydrogen peroxide. Subsequently, sections were incubated with the diluted (1∶200 in PBS, Biolegend, USA) biotinylated mouse antihuman FOXP3 antibody in a humidified chamber at 4°C overnight. Thereafter, the sections were incubated with streptavidin conjugated horseradish peroxidase (Zymed, USA) for 30 minutes, followed by development with 3,30-diaminobenzidine (Zymed) for 5 minutes, and counter-staining with hematoxylin. Negative control use appropriate serum for the primary antibody. Positive and negative controls were used for all immunohistochemical stains. Images were captured on a Leica DM 4000 B microscope (Solms, Germany).

Foxp3 immunostaining was scored by two independent observers without knowing the group of the section. Ten fields per subject were semiquantitatively scored in each section as follows: 0, no staining; 1, weak staining; 2, moderate staining; and 3, strong staining [7].

### Statistical Analysis

Data were expressed as the mean ± standard deviation (SD) or number (%). Clinic-pathological characteristics were compared using the Student’s t-test and Chi-square test. Genotype frequencies of the two *Foxp3* polymorphisms were tested for deviation from the Hardy–Weinberg equilibrium by using Chi-square test (http://oege.org/software/hardy-weinberg.shtml). Comparison of the allele and genotyping frequencies between case and control was performed by Chi-square test. Odds ratios (OR) and 95% confidence intervals (CI) were calculated to assess the disease risk conferred by a particular genotype or allele. Multivariate logistic regression was carried out with adjustment for potential confounding covariates (maternal age, nulliparity, and gestational age at delivery week). *P*<0.05 was considered as statistical significance. Statistical analysis was performed using the Statistical Package for Social Sciences version 10.0 (SPSS Inc., Chicago, IL, USA).

## Results

### Distribution of Demographic and Clinic-pathological Characteristics

The distribution of demographic and clinic-pathological characteristics for PE and healthy controls were showed in Table 1. The median ages of healthy controls and PE women were, respectively, 28.3 (SD = 4.5) and 30.5 (SD = 3.9) years,. In PE group, the fetal birth weight was significantly lower, and the gestational age at delivery was markedly shorter, whereas proteinuria, nulliparity and blood pressure were significantly higher than that in control group.

**Table 1:** Demographic and clinic-pathological characteristics of subjects (Mean±SD).

Characteristics	PE (n = 156)	Control (n = 252)
Age(y)	30.5±4.5	28.3±3.9
GA at delivery (wk)	33+3	38+6
Family history of hypertension	16 (14.95%)	2 (0.77%)*
Preterm	86 (80.37%)	18 (6.90%)*
Cesarean section	79 (73.83%)	116 (44.44%)
Birth weight	1821.48	3186.30 #
Placental weight (g)	645.4±70. 0	723.3±66. #
Bp	164.89	134.67*
proteinuria	3.986	0.284*

* Compared with control,

P<0.05;

# Compared with control, P<0.01.

### Foxp3 Expression Changes in Decidual and Trophoblast Cells

**Figure 1:**
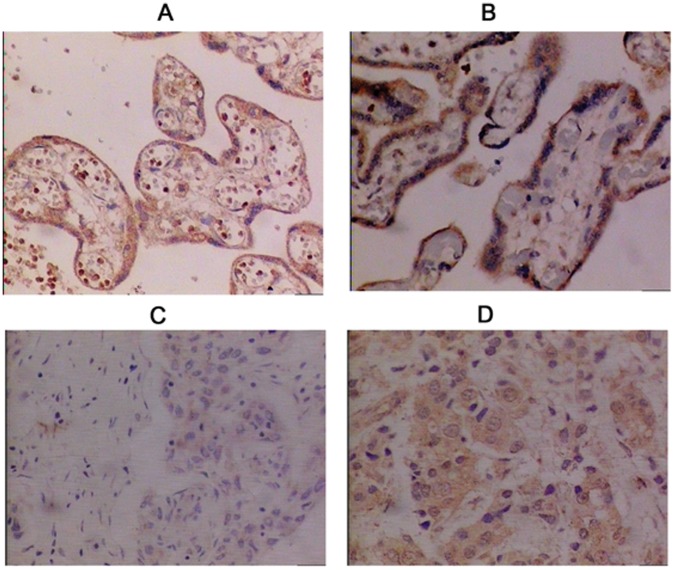
Placental expression of Foxp3 in normal and PE groups. Figure 1A PE group placental trophoblast showed Foxp3 positive cells in immunohistochemical staining × 200. Figure 1B Control group placental trophoblast showed Foxp3 positive cells in immunohistochemical staining × 200. Figure 1C PE group placenta decidua showed Foxp3 positive cells in immunohistochemical staining × 200. Figure 1D Control group decidual cells showed Foxp3 positive cells by immunohistochemical staining × 200.

The expression of Foxp3 in placenta is similar between PE and control groups (Figure 1). The tinting strength in PE group was significantly enhanced indicating Foxp3 expression was significantly higher compared with controls. The positive rates of Foxp3 expression in the decidua of pregnant women were, respectively, 16.67% and 66.67% for PE and control groups, suggesting Foxp3 expression was significantly lower in PE than that in control (*P*<0.05, Table 2).

**Table 2:** Foxp3 expression levels in decidual and trophoblast cells between PE and control groups.

Group case number	Foxp3 expression				Positive rate (%)
	−	+	++	+++	
PE (n = 30)	22	7	1	0	26.67
Control (n = 30)	11	9	8	2	63.33*

* Compared with control, P<0.05.

### Identification of Foxp3-3279 Polymorphism

**Figure 2:**
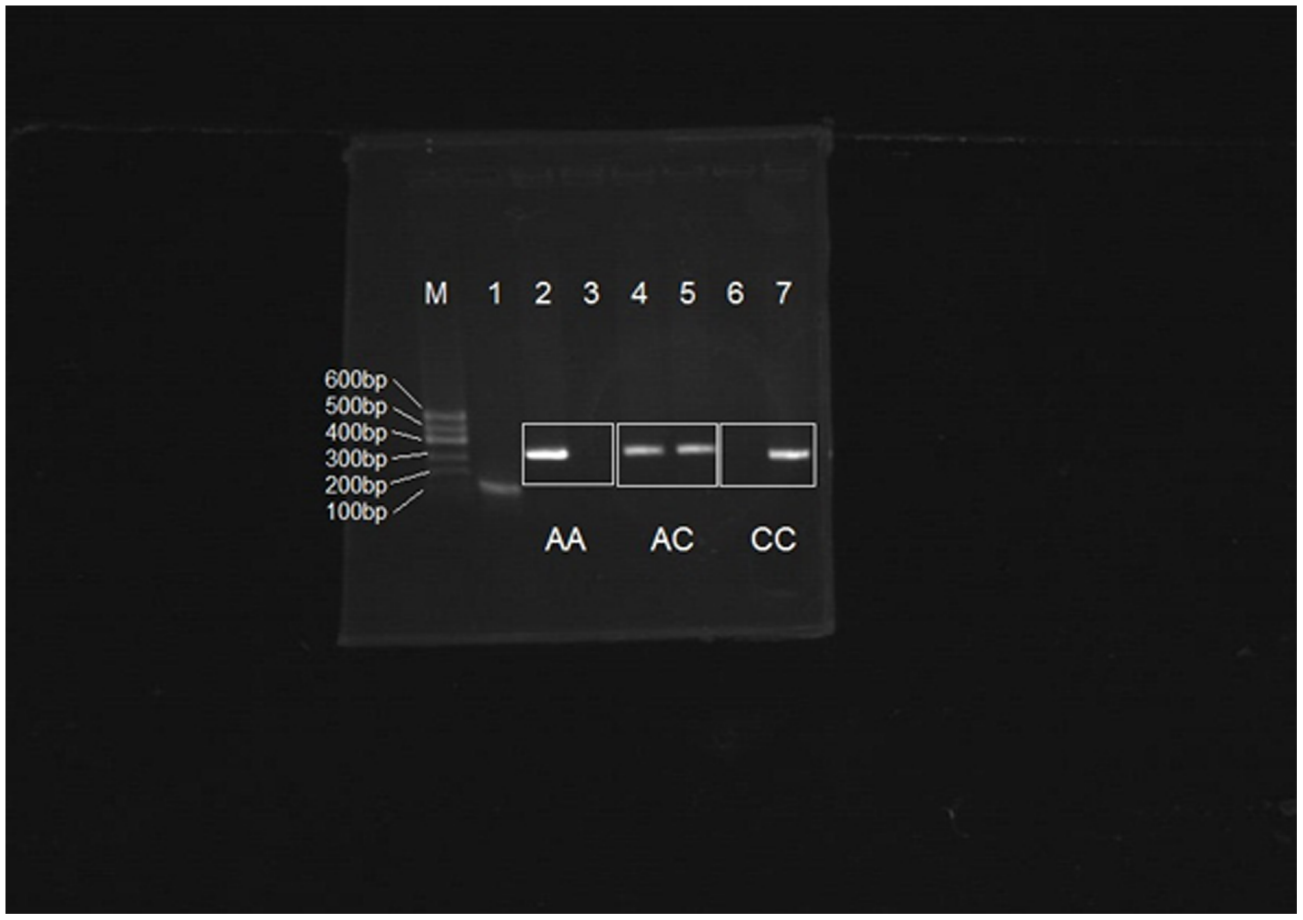
Foxp3-3279 C allele in Agarose gel electrophoresis. Lane 1 showed the β-Actin, Lane 2 and 4 showed the positive PCR products amplified by group A primer (F1 primer); Lane 6 showed the negative PCR products amplified by group A primer. Lane 5 and 7 showed the positive PCR products amplified by group C primer (F2 primer); Lane 3 showed the negative PCR products amplified by group C primers; Lane M:DNA marker.

**Figure 3:**
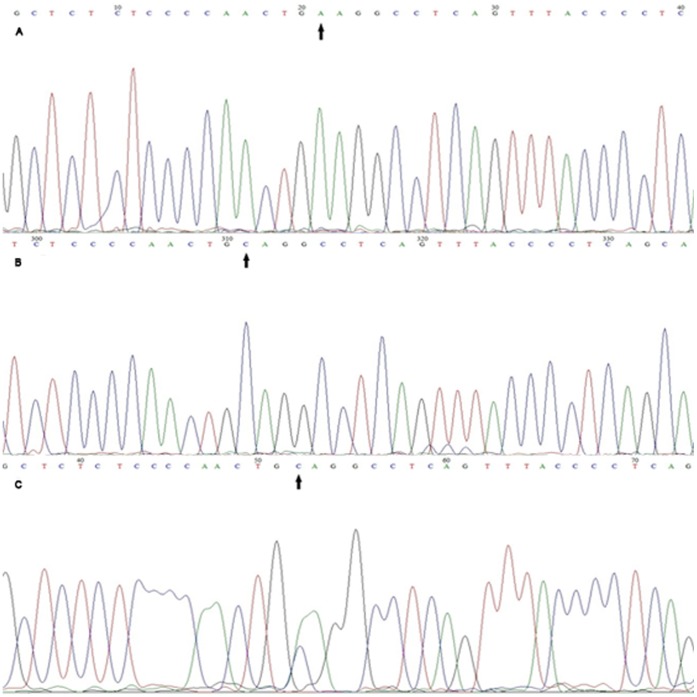
Sequencing results of *Foxp3*-3279. Figure 3A was the sequencing of *Foxp3*-3279A/A genotype; Figure 3B was the sequencing of *Foxp3*-3279C/C genotype. Figure 3C was the sequencing of *Foxp3*-3279 A/C genotype. The arrow part was the SNP sites.

The PCR amplification product of Foxp3-3279 target is 333 bp. Ten percent of the samples in PE and control groups were selected to repeat PCR amplification five times and obtained the same results. Foxp3-3279 allele specified: CC genotype is only visible using group C primer (F2 primer) but not in group A primer (F1 primer); AA genotype is only visible using group A primer; and AC genotype is observed in two sets of primers (Figure 2).And Figure 3 showed the DNA sequencing result of Foxp3-3279.

### Identification of Foxp3-6054 Polymorphism

**Figure 4:**
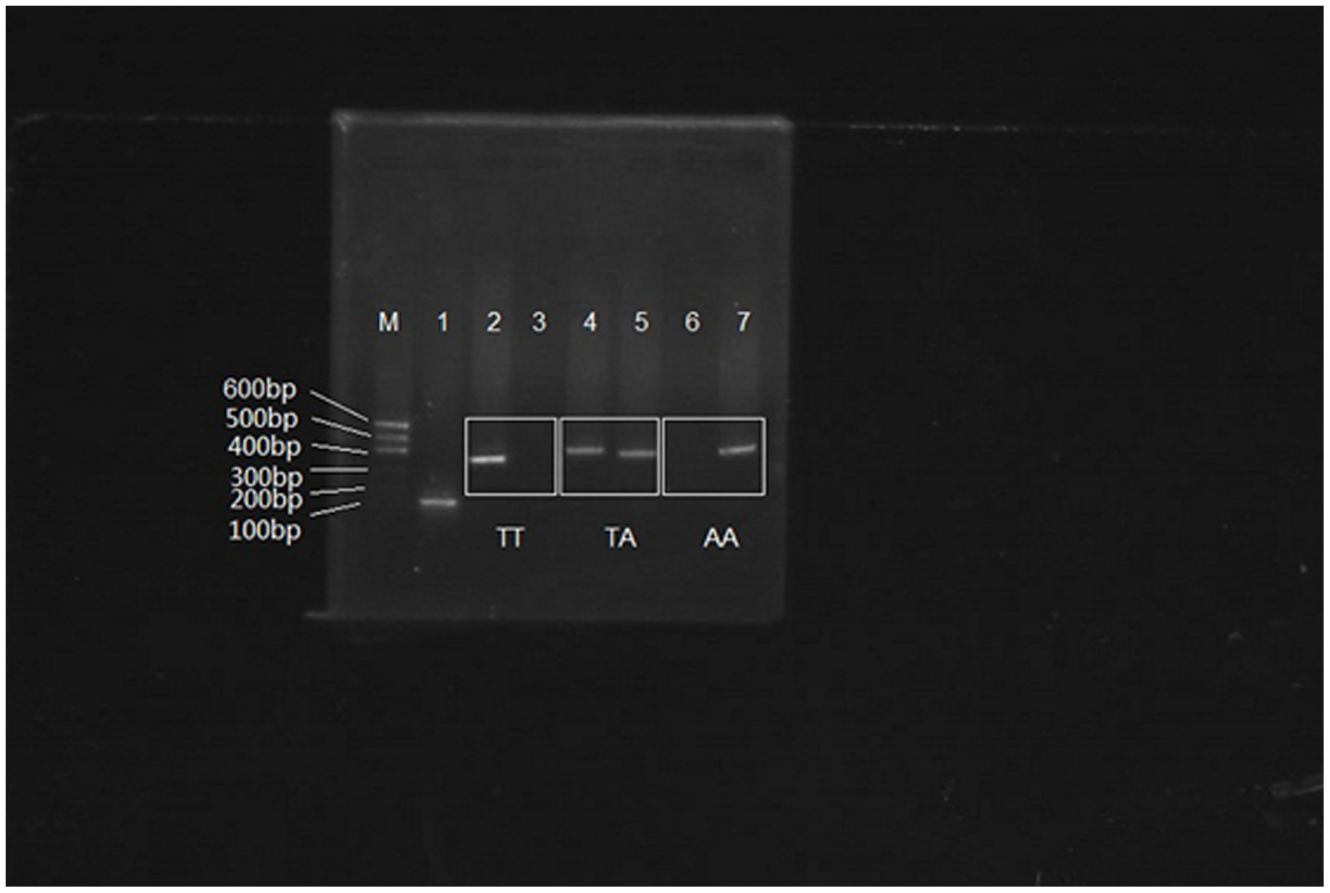
Foxp3-6054 ATT allele in Agarose gel electrophoresis. Lane 1 showed the β-Actin, Lane 2 and 4 showed the positive PCR products amplified by group del primer (F1 primer); Lane 6 showed the negative PCR products amplified by group del primer. Lane 5 and 7 showed the positive PCR products amplified by group ATT primer (F2 primer); Lane 3 showed the negative PCR products amplified by group ATT primers; Lane M: DNA marker.

**Figure 5:**
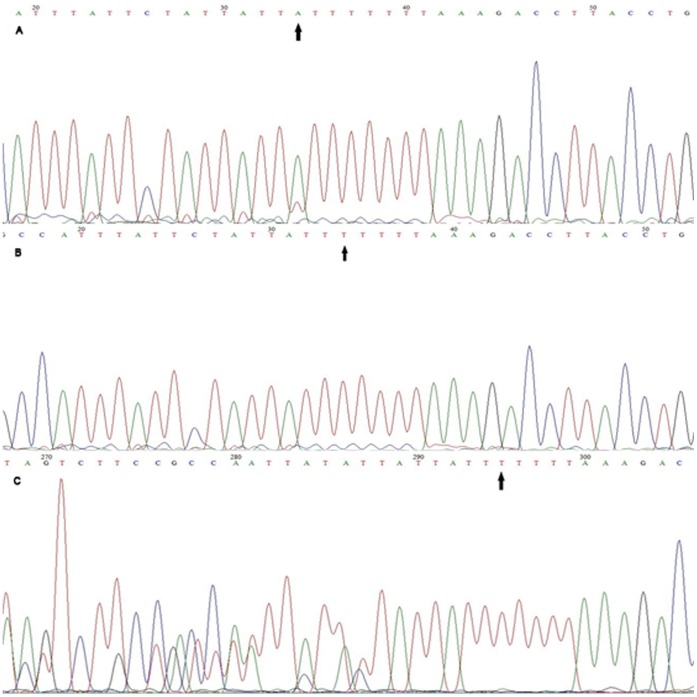
Sequencing results of *Foxp3*-6054. Figure 5A was the sequencing of *Foxp3*-6054 A/A genotype; Figure 5B was the sequencing of *Foxp3*-6054 T/T genotype. Figure 5C was the sequencing of *Foxp3*-6054 T/A genotype. The arrow part was the SNP sites.

The PCR amplification product of Foxp3-6054 target is 442 bp. Ten percent of the samplesin PE and control groups were selected to repeat PCR amplification five times and got the same results. Foxp3-6054 TT (del/del) genotype is only visible in group T primer (F1 primer) amplified PCR products; genotype AA (ATT/ATT) is only visible in group A primer (F2 primer) amplified PCR products, and TA (del/ATT) genotype is visible in both group T and A primers amplified PCR products (Figure 4). And Figure 5 showed the DNA sequencing result of Foxp3-6054.

### Foxp3-6054, -3279 Allele and Genotype Frequencies in PE and Control Groups

Genetic polymorphisms analyses of Foxp3-6054 and -3279 were performed in 156 PE patients and 252 healthy controls. The distributions of detected genotypes were compatible with those expected from the Hardy-Weinberg equilibrium (P>0.05), indicating the balance of population genetic data from the same Mendelian population.

The genotypic and allelic frequencies were shown in Table 3. The genotyping frequencies of Foxp3-3279 were, respectively, 73.08% for CC, 24.64% for CA and 1.28% for AA in PE group. Similar trend is observed in control group. No significant difference for Foxp3-3279 genotyping was obtained between PE and control groups (P>0.05, Table 3).

**Table 3:** Allele and genotyping frequencies of Foxp3-3279 and -6054 in PE and control groups.

Group	No.	–3279Genotype (%)			–3279Allele (%)		–6054Genotype (%)			–6054Allele (%)	
		C/C	C/A	A/A	C	A	T/T	T/A	A/A	T	A
PEControl	156	114(73.08%)	40(24.64%)	2(1.28%)	0.8589	0.1411	50(32.05%)	81(51.92%)	25(16.02%)	0.4199	0.5802
	252	184(73.02%)	62(24.60%)	6(2.38%)	0.8532	0.1468	110(43.65%)	114(45.24%)	28(11.11%)	0.3373	0.6627
X^2^		0	0.06		0.05	0.05	5.44	1.73	2.89	5.64	5.64
p-value		P>0.05	P>0.05	p = 0.23	P>0.05	P>0.05	p<0.05	p>0.05	p>0.05	p<0.05	p<0.05
RR		1.001	1.042	0.538	1.007	0.873	0.7342			0.875	1.245
95%CI		1.1098	1.4679	2.6347	1.0138	1.2327	0.9607			0.9828	1.4906
		0.9012	0.7394	0.1101	1.0023	0.618	0.5615			0.7799	1.0394

Logistic regression analysis indicated that Foxp3-6054 TT genotype was significantly associated with reduced risk of PE (P<0.05, RR = 0.73 95% CI: 0.56–0.96, Table 3). A non-significant increased risk was observed for AA genotype (Table 3). A dangerous trend to increase the risk of pregnancy-induced hypertension was observed (data not shown). The merger genotype of AT/AA significantly increased the risk of suffering from preeclampsia (RR = 1.21, 95%CI: 1.03–1.40, Table 4).

**Table 4:** RR and 95%CI of Foxp3 -6054 AT+AA mergergenotype in PE and control groups.

	AT+AA	TT	Total
PE	106	50	156
Control	142	110	252
Total	248	160	408
X^2^	5.44	5.44	
p	p<0.05	p<0.05	
RR	1.2058	0.7342	
95%CI	1.4049	0.9598	
	1.0348	0.5617	

### Foxp3 Haplotypes and PE Risk

The haplotype analyses for Foxp3-6054 and -3279 polymorphisms found that 6054A/−3279C haplotype is significantly associated with increased PE risk (p<0.05, RR = 2.35, 95% CI: 1.01–5.42, Table 5), while -6054T/−3279C is significantly associated with reduced PE risk (p<0.05, RR = 0.75, 95% CI: 0.57–0.9979, Table 5). No other haplotype frequency has significant differences between PE and control groups (Table 5).

**Table 5:** Foxp3-6054/−3279 haplotype frequency in PE and Control groups.

Foxp3-6054	Foxp3-3279	Control groups	PE	X2	p	RR	95%CI	
Foxp3-6054 del/del	Foxp3-3279 C/C	108	50	4.74	p<0.05	0.7479	0.9979	0.5711
Foxp3-6054 del/ATT	Foxp3-3279 C/C	65	48	1.04				
Foxp3-6054 ATT/ATT	Foxp3-3279 C/C	11	16	5.41	p<0.05	2.3497	5.4207	1.0118
Foxp3-6054 del/del	Foxp3-3279 C/A	1	0		p = 0.62			
Foxp3-6054 del/ATT	Foxp3-3279 C/A	46	32	3.84		1.1237	1.6839	1.3323
Foxp3-6054 ATT/ATT	Foxp3-3279 C/A	15	8	0.12				
Foxp3-6054 del/del	Foxp3-3279 A/A	1	0		p = 0.62			
Foxp3-6054 del/ATT	Foxp3-3279 A/A	3	1		p = 0.36			
Foxp3-6054 ATT/ATT	Foxp3-3279 A/A	2	1		p = 0.44			

## Discussion

Human *Foxp3* gene located at chromosome Xp11 containing 11 exons and 10 introns 23. *Foxp3* is not only a sign of CD4 CD25 Treg cells activation, but also correlated with cells’ functions (development and maintenance of cells) in human. Previous studies [12] have found 13 mutations in human *Foxp3* gene and are associated with autoimmune diseases Park et al [3] found four *Foxp3* SNPs in Crohn's disease with an increased risk for homozygous genotyping, Yang et al [4] demonstrated that *Foxp3* -6054 and -3279 polymorphisms were associated with the onset of alopecia areata. Other studies found *Foxp3* -6054 and -3279 polymorphisms were associated with habitual abortion [5], systemic lupus erythematosus [6] and allergic rhinitis [7]. Shen et al [8] confirmed that Foxp3 -6054 and -3279 polymorphisms can inhibit the transcription of Foxp3, thereby reduce the expression of non-active Treg cells. Foxp3 down-expression in PE has been reported in many previous studies [14]-[16], but the mechanism involved in is still unclear. Whether *Foxp3* mutations play a role in regulating Foxp3 expression need further investigation.

Our results indicate that *Foxp3*-6054 TT genotype was significantly associated with reduced PE risk compared with AA genotype. Combination of AT/AA was also significantly associated with increased PE risk. *Foxp3* -3279 is not associated with PE risk. Our data provided more evidence to support the potential role of *Foxp3* polymorphisms in PE susceptibility. Case-control study including 120 women with preeclampsia and 120 healthy normotensive controls was conducted. Genetic variants (single nucleotide polymorphisms and microsatellites) in the FOXP3 gene were analyzed in the USA, Torri D,et al [9] found that there were no differences in the genotypes or allele frequencies for the SNPs including rs6609857, rs2294020, rs2280883, rs2232367, rs3761547, and rs4824747 except *Foxp3*-6054 and -3279 between cases and controls. The FOXP3 GT microsatellite allele at 266 bp was less common in cases than that in controls (1.0% vs 5.2%, P = 0.0264). Jahan P, et al [10] found that A allele -3279 to be protective against PE and C allele -3279 as predisposing in a dose dependent manner in india population. Hassannia [11] and André et al. [12] found that *Foxp3*-6054 and -3279 haploid were associated with diseases risk, suggesting that the impact of gene on disease risk may not limit to single SNP. The analysis of haplotype or genes’ interaction should also pay attention. Consistent with this observation, we also found that PE was significantly increased in Foxp3-6054A/−3279C haplotype frequency compared with control (p<0.05), while -6054T/−3279C haplotype frequency was significantly reduced in PE (p<0.05).

Our current findings are biological plausible. During the normal pregnancy, the fetus usually decreased the rejection immune response and enhanced the protective effect of immune response [17]. While PE-related immune system changes display an opposite response, i.e. enhanced fetal rejection and reduced protective reaction. One study found that the peripheral lymphocytes of PE stimulate the stepmother cell’s response and decrease their spouses’ lymphocytes. The reaction of mixed lymphocyte culture was decreased in macrophages of PE with enhanced NK cell activity. These macrophages are activated again after the release of cytokines involved in vascular endothelial cells [17]. Another anti-endometrial antibody from PE was increase in circulating immune complexes, complement activation, and immune complex deposition in spiral arteries of the uterus, placenta, liver, kidney and skin [18]. Th1/Th2 ratio changes, TCR chain, CD3 decline, increased proinflammatory and cytokines gathered were also observed [19]. Change of these cytokines is a direct result of vascular endothelial cells damage caused by imbalance of vasoactive substances. It may lead to high blood pressure, edema, proteinuria, and a series of clinical manifestations of pregnancy-induced hypertension. Therefore, immune disorders or immune response has important link with PE. Epidemiological studies found that PE patients exhibited a familial tendency, i.e. the risk of first-degree relatives of PE was significantly higher, indicating genetic factors play an important role in PE susceptibility. Therefore, we believe that PE is a polygenic disease influenced by a variety of environmental and genetic factors. *Foxp3* polymorphisms may be one of the factors involved in PE susceptibility.

Wang et al. [1] detected CD4 and CD25 in the peripheral blood and umbilical cord blood of PE patients. Foxp3 expression in Tregs was significantly lower than normal pregnancy and the newborns. This may result in maternal-fetal immune tolerance imbalance, and lead to the occurrence of PE. Cao et al. [2] found that the CD4/CD25 ratio and the expression of Foxp3 in CD4, CD25 were significantly lower in mild and severe PE patients than that in normal pregnant women, suggesting the changes of regulatory T cells involved in the pathological process of hypertensive disorders during pregnancy. Placenta as a fetal mother interface plays a similar role. In the current study, we observed 30 PE patients had placental Foxp3 expression, but the expression level of Foxp3-positive cell is lower than normal pregnancy control. This preliminary data indicated that placental Foxp3 expression may be one of the mechanisms involved in maternal immune tolerance to embryo antigen, and further impact on the pathologic process of PE.

Our study found that *Foxp3*-6054 TT genotype and -6054T/−3279C haplotype were significantly associated with reduced PE risk, whereas AT/AA mergergenotype and -6054A/−3279C haplotype significantly increased PE risk. We concluded that these genotypes may contribute to the susceptibility of preeclampsia. However, the detailed pathogenic role of Foxp3 in PE is still unclear. Functional study is necessary in the future.

## References

[1] Wang LL, Cao XW (2010) Patients with preeclampsia in peripheral blood and umbilical CD +4 CD +25 of Foxp3+ of Treg levels. Shandong Medical Journal 50 (26): 10.

[2] Cao WP, Qian QJ, Jian W (2010) Changes and significance of the peripheral blood CD4+ CD25+ Foxp3+ regulatory T cell in gestational hypertension patients. Pathophysiology 26 (7): 1425–1427.

[3] Park O, Grishina I, Leung PS, Gershwin ME, Prindiville T (2005) Analysis of the Foxp3/scurfin gene in Crohn's disease.Ann N Y Acad Sci. 1051: 218–28. 10.1196/annals.1361.125 16126962

[4] Yang SY, Guo X, Song ZQ (2010) FOXP3 gene polymorphism and alopecia areata association studies. Journal of Immunology 26 (4): 320.

[5] Wu Z, You Z, Zhang C, Li Z, Su X, et al (2012) Association between functional polymorphisms of Foxp3 gene and the occurrence of unexplained recurrent spontaneous abortion in a Chinese Han population. Clin Dev Immunol 2012: 896458. 21876709 10.1155/2012/896458 PMC3162971

[6] Lin YC, Lee JH, Wu AS, Tsai CY, Yu HH, et al (2011) Association of single-nucleotide polymorphisms in FOXP3 gene with systemic lupus erythematosus susceptibility: a case-control study. Lupus 20(2): 137–43. 21078762 10.1177/0961203310382428

[7] Fodor E, Garaczi E, Polyánka H, Koreck A, Kemény L, et al (2011) The rs3761548 polymorphism of FOXP3 is a protective genetic factor against allergic rhinitis in the Hungarian female population. Hum Immunol Oct 72(10): 926–9. 10.1016/j.humimm.2011.06.011 21763379

[8] Shen Z, Chen L, Hao F, Wang G, Liu Y (2010) Intron-1 rs 3761548 is related to the defective transcription of foxp3 in psoriasis through abrogating E47/c-Myb binding. J Cell Mol Med Jan 14(1–2): 226. 10.1111/j.1582-4934.2008.00370.x PMC3837602 20414968

[9] Metz TD, Nelson LM, Stoddard GJ, Silver RM (2012) FOXP3 gene polymorphisms in preeclampsia. Am J Obstet Gynecol Feb 206(2): 165.e1–6. 10.1016/j.ajog.2011.09.005 22000667

[10] Jahan P, Sreenivasagari R, Goudi D, Komaravalli PL, Ishaq M (2013) Role of Foxp3 Gene in Maternal Susceptibility to Pre-eclampsia - A Study From South India. Scand J Immunol Feb 77(2): 104–8. 10.1111/j.1365-3083.2012.02760.x 22809231

[11] Hassannia H, Abediankenari S, Ghaffari J (2011) FOXP3 and TGF-β Gene Polymorphisms in Allergic Rhinitis. Iran J Immunol Dec 8(4): 218–25. 10.22034/iji.2011.17029 22201619

[12] André GM, Barbosa CP, Teles JS, Vilarino FL, Christofolini DM, et al (2011) Analysis of FOXP3 polymorphisms in infertile women with and without endometriosis. Fertil Steril Jun 95(7): 2223–7. 10.1016/j.fertnstert.2011.03.033 21481380

[13] Fontenot JD, Rasmussen JP, Williams LM, Dooley JL, Farr AG, et al (2005) Regulatory T cell lineage specification by the forkhead transcription factor foxp3. Immunity Mar 22(3): 329–41. 10.1016/j.immuni.2005.01.016 15780990

[14] Santner-Nanan B, Peek MJ, Khanam R, Richarts L, Zhu E, et al (2009) Systemic Increase in the Ratio between Foxp3^+^ and IL-17-Producing CD4^+^T Cells in Healthy Pregnancy but not in Preeclampsia. J Immunol Dec 1 183(11): 7023–30. 10.4049/jimmunol.0901154 19915051

[15] Toldi G, Svec P, Vásárhelyi B, Mészáros G, Rigó J, et al (2008) Decreased number of FoxP3+ regulatory T cells in preeclampsia. Acta Obstet Gynecol Scand 87(11): 1229–33. 19016357 10.1080/00016340802389470

[16] Quinn KH, Lacoursiere DY, Cui L, Bui J, Parast MM (2011) The unique pathophysiology of early-onset severe preeclampsia: role of decidual T regulatory cells. J Reprod Immunol Sep 91(1–2): 76–82. 10.1016/j.jri.2011.05.006 21782252

[17] Irani RA, Zhang Y, Zhou CC, Blackwell SC, Hicks MJ, et al (2010) Autoantibody-mediated angiotensin receptor activation contributes to preeclampsia through TNF-alpha signaling. Hypertension May 55(5): 1246–53. 10.1161/HYPERTENSIONAHA.110.150540 PMC3380607 20351341

[18] Ramma W, Ahmed A (2011) Is inflammation the cause of pre-eclampsia? Biochem Soc Trans Dec 39(6): 1619–27. 10.1042/BST20110672 PMC3222326 22103497

[19] Vitoratos N, Hassiakos D, Iavazzo C (2012) Molecular Mechanisms of Preeclampsia. J Pregnancy 2012: 298343. 22523688 10.1155/2012/298343 PMC3317114

