# Correction: *Foxp3* (−/ATT) Polymorphism Contributes to the Susceptibility of Preeclampsia

**DOI:** 10.1371/annotation/8d966a21-a3a3-448a-b29f-a843674b8c49

**Published:** 2013-08-13

**Authors:** Ximing Chen, Ting Gan, Zhiqiong Liao, Shengqiang Chen, Jianhua Xiao

Institutional affiliation 1 for authors Ximing Chen and Jianhua Xiao is incorrect. The correct affiliation is: 1. Medical College of University of South China， Hengyang, China. 

In addition, the Corresponding Author for the article was incorrectly listed as Ximing Chen. The Corresponding Author is Jianhua Xiao, who can be reached at JianhuaXia2010@163.com

